# The Role of Nuclear Medicine for COVID-19: Time to Act Now

**DOI:** 10.2967/jnumed.120.246611

**Published:** 2020-06

**Authors:** Freimut D. Juengling, Antonio Maldonado, Frank Wuest, Thomas Hellmut Schindler

**Affiliations:** * University Bern Murtenstrasse 11 Bern, Switzerland 3008 E-mail: freimut.juengling@med.unibe.ch

**TO THE EDITOR:** As every medical department worldwide is bracing for the impact the coronavirus disease 2019 (COVID-19) pandemic will pose on daily routines, the scientific community starts to excel in exploring the different facets this novel disease bears for prevention, diagnosis, and treatment. Nuclear medicine, touting itself “molecular medicine” as well as “theranostic medicine” may not fall short here, as we have much to offer.

Although it may be wise for individual nuclear medicine departments to focus on taking precautions for themselves and their most vulnerable patients, with oncologic and cardiovascular or neurologic “predispositions,” to the risks of exposure to COVID-19–bearing individuals (1), we should still motivate ourselves and the academic nuclear medicine community to actively participate in improving care for COVID-19 patients.

Accumulating evidence suggests that some of the detrimental effects seen in patients with severe COVID-19 are attributed to an overly host antiviral defense as seen in severe acute respiratory syndrome (SARS), leading to hyperinflammatory reactions or cytokine storm syndrome, sometimes also affecting the central nervous system (CNS) (2,3).

Until now, however, existing knowledge regarding supportive care and adjunctive pharmacologic therapy is limited. Even worse, a subgroup of COVID-19 patients that seems to do well after getting out of the intensive care unit dies of acute respiratory syndrome just several days later, without clinical signs indicating their imminent deterioration. This situation may be one of the first places where nuclear medicine should tune in: With ^18^F-FDG PET/CT for decades being well evaluated for its sensitivity in detecting inflammatory disease (4), we should start to prospectively collect data in a well-defined group of patients at given time intervals during the course of COVID-19 infection to better understand the inflammatory component of the disease and maybe find early prognostic signs that warrant proactive antiinflammatory treatment in patients at risk. Until now, only anecdotal data exist (5). As a nuclear medicine community, we ought to team up and establish protocols suitable for multicentric evaluation as soon as possible, meeting the requirements for controlled trials. Serial examinations should include both cohorts of patients with proven COVID-19 with different severity and extended follow-up after recovery as well as symptomatic patients with radiographic findings typical for COVID-19, but without initial proof of infection, and should not only focus on pulmonary inflammation but also address possible inflammatory involvement (e.g., of myocardium, pericardium, vasculature, muscles, intestine, and the CNS.

At academic sites providing research facilities including cyclotron and radiopharmacy production, the research into the inflammatory cascade could go even further. There are well-established radiopharmaceuticals suitable as inflammatory biomarkers at an intracellular level, for example, targeting the purinergic P2X7 receptor (6). Although initially designed to quantify neuroinflammation, they can easily be repurposed for imaging the inflammasome and quantifying inflammation at a whole-body level. As there are also potential P2X7 inhibitors at the receptor level (6), showing antiinflammatory effects in animal models (7), translational research may here form the rationale for antiinflammatory therapy principles in COVID-19. Furthermore, nuclear medicine has the potential to provide evidence and clarify contradictory concepts in the use of nonsteroidal antiinflammatory drugs in COVID-19, where clinicians have to state, “many clinical anecdotes remain stalled in biologic plausibility” (8), by directly depicting cyclooxygenase-2 (COX2) involvement using established COX2-inhibitory radiopharmaceuticals (9).

Possible repurposing of other established radiopharmaceuticals to investigate COVID-19–specific pathomechanisms might target the cytokine signaling pathway (e.g., chemokine receptor CXCR4, interleukin IL-6), fibroblast activation protein inhibitors (FAPI), to address postinflammatory fibrosis, or inhibitors of the type 1 angiotensin-II-receptor ATR1 (e.g., KR31173), involved in cellular internalization of SARS-CoV-2 (2). Development of novel radiopharmaceuticals could also focus on directly targeting the entry receptor for SARS-CoV-2, the angiotensin-converting-enzyme-2 (ACE2). Radiolabeling of an ACE2-receptor antagonist has already been achieved for receptor autoradiography protocols (10) and could serve as a starting point for PET tracer development, increasing our readiness for the next coronavirus shift.

The recent World Health Organization initiative of creating a voluntary intellectual property pool for COVID-19 products to balance intellectual property and accessibility could address some of the issues inhibiting broader application of new tracers. Regulatory agencies also recently have shown extraordinary performance in overviewing new applications. And nuclear medicine already has proven in many ways to excel in logistics for radiopharmaceutical distribution, partnering with academic, administration, and industrial stakeholders.

The examples chosen here are not meant to be comprehensive. They are meant to stimulate our community’s potential to contribute to one of the biggest challenges in modern medicine. The existing Society of Nuclear Medicine and Molecular Imaging Connect platform may serve as a natural vehicle to collectively define and distribute protocols suitable for multicentric trials and to share what it needs for making novel radiopharmaceuticals accessible at an accelerated time scale.

Let us build a network of clinical trials, let us start now and fast and bold. It is time to act now.

## DISCLOSURE

No potential conflict of interest relevant to this article was reported.
